# Golden Gate Assembly system dedicated to complex pathway manipulation in *Yarrowia lipolytica*

**DOI:** 10.1111/1751-7915.12605

**Published:** 2017-02-19

**Authors:** Ewelina Celińska, Rodrigo Ledesma‐Amaro, Macarena Larroude, Tristan Rossignol, Cyrille Pauthenier, Jean‐Marc Nicaud

**Affiliations:** ^1^ Department of Biotechnology and Food MicrobiologyPoznan University of Life Sciencesul. Wojska Polskiego 4860‐627PoznanPoland; ^2^ Micalis InstituteINRA, AgroParisTech, Université Paris‐Saclay78350Jouy‐en‐JosasFrance; ^3^ Institute of System and Synthetic BiologyUniversite d'Evry vald'EssonnesBt. Geneavenir 6 Genopole Campus 1, 5 rue Henry Desbrueres91000EvryFrance

## Abstract

In this study, we have adopted Golden Gate modular cloning strategy to develop a robust and versatile DNA assembly platform for the nonconventional yeast *Yarrowia lipolytica*. To this end, a broad set of destination vectors and interchangeable building blocks have been constructed. The DNA modules were assembled on a scaffold of predesigned 4 nt overhangs covering three transcription units (each bearing promoter, gene and terminator), selection marker gene and genomic integration targeting sequences, constituting altogether thirteen elements. Previously validated DNA modules (regulatory elements and selection markers) were adopted as the Golden Gate bricks. The system's operability was demonstrated based on synthetic pathway of carotenoid production. This technology greatly enriches a molecular biology toolbox dedicated to this industrially relevant microorganism enabling fast combinatorial cloning of complex synthetic pathways.

## Introduction

Synthetic biology, and in particular combinatorial cloning, derives from engineering concepts of standardization, modularity of building blocks and simplification of assembly lines. Traditional protocols employing restriction digestion and one‐by‐one element cloning are both time‐ and cost‐inefficient (Celińska and Grajek, 2013; Matthaus *et al*., 2014). Due to the recent development of DNA assembly techniques for metabolic pathway engineering, a great worldwide effort is now being pursued towards establishing such cloning platforms for an individual organism of interest. In the last years, several DNA modular assembly platforms have been developed and new standards have been defined (Sands and Brent, 2016). Golden Gate (GG) modular cloning system, relying on type IIs restriction enzymes, appears as one of the most robust techniques within this field (Engler *et al*., 2008; Gao *et al*., 2013). The core of GG strategy lies in establishing a library of standardized and interchangeable DNA parts, which can be subsequently assembled in a single‐step, one‐pot reaction. Examples of such GG platforms have been recently reported for *Escherichia coli*, yeast or plant species (Engler *et al*., 2014; Terfrüchte *et al*., 2014; Agmon *et al*., 2015; Kakui *et al*., 2015; Lee *et al*., 2015; Mitchell *et al*., 2015; Iverson *et al*., 2016; Moore *et al*., 2016). Nevertheless, many of valuable biotech workhorses are still queuing the line for a customized GG platform, amongst these the non‐conventional yeast *Y. lipolytica*, which is a well‐established biotechnological chassis for the production of numerous valuable bioproducts (Nicaud, 2012; Ledesma‐Amaro *et al*., 2015; Madzak, 2015; Ledesma‐Amaro and Nicaud, 2016). Establishing a GG combinatorial cloning platform for *Y. lipolytica* appears as an urgent need, enabling more time‐ and cost‐efficient assembly of complex DNA constructions and standardization of DNA modules, which can be easily exchanged between different laboratories.

Here, we present a robust modular GG system for expression of one, two or three customizable transcription units (TU) in a versatile cassette for *Y. lipolytica*.

## Results and discussion

### Defining standards and modular parts of the GG system for *Y. lipolytica*

**Figure 1:**
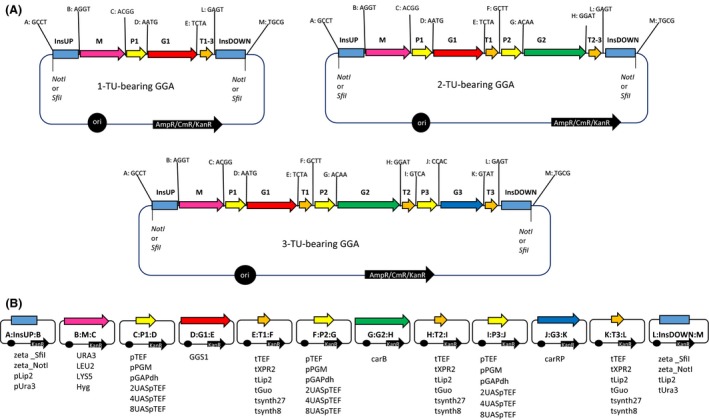
Golden Gate Assembly platform dedicated to *Yarrowia lipolytica* in a one‐, two‐ and three‐transcription unit (TU)‐bearing format. A. Schematic representation of complete GG destination vectors in a one‐, two‐ and three‐TU‐bearing format. Each of GGA covers at least one TU (composed of promoter – P; gene as ORF – G; and terminator – T) and selection marker (M), which are flanked with integration targeting sequences (InsUP and InsDOWN), constructed on a destination vector backbone, either pSB1A/C/K3‐RFP/amilCP/amilGFP for ampicillin (A), chloramphenicol (C) or kanamycin (K) resistance marker respectively; pSB vector series offers exploitation of one of the following reporter genes in *E. coli*: RFP – red chromophore; amilCP – blue chromophore; amilGFP – green fluorophore. Building blocks are flanked with predesigned 4 nt overhang, each assigned a letter from A–M. Sequences of the overhangs are provided next to the respective position. Abbreviations: InsUP/DOWN – sequence targeting insertion in the 5'/3' region; M – selection marker; P1/2/3 – promoter governing expression of the gene of interest in transcription unit 1, 2 and 3 respectively; G1/2/3 – gene of interest in transcription unit 1, 2 and 3 respectively; T1/2/3 – terminator located in the transcription unit 1, 2 and 3 respectively. NotI/SfiI: NotI/SfiI recognition sites for release of the expression cassettes prior to *Y. lipolytica* transformation. B. Donor vectors constructed on a backbone of pCR II‐Blunt TOPO vector (Invitrogen/Life Technologies) bearing kanamycin resistance gene. Each building block is flanked with respective BsaI recognition sites and predesigned 4 nt overhangs. Exemplary building blocks (Golden Gate Fragments; GGFs) constructed in this study are provided as a list underneath the donor vectors' schemes. For reference to nucleotide sequences, see Supporting Information.

The ultimate goal of this study was the development of extensive and versatile system of building blocks (Golden Gate Fragments; GGFs) easily fitted into the general scaffold of GGA (Golden Gate Assembly), enabling fast construction of complex expression vectors dedicated to metabolic engineering of *Y. lipolytica*. To complete this task, a set of thirteen 4 nt overhangs was developed together with the corresponding destination vector system (Fig. 1, Supporting Information) to cover three TUs (all composed of promoter – P; ORF = gene – G; terminator – T; all accompanied with a suffix indicating position of a TU); selection marker gene – M; and integration targeting sites – InsUP and InsDOWN.

Incompatibility of the 4 nt sequence was a prerequisite to avoid the risk of unintended matching of the building blocks in uncontrolled position and/or orientation. Such a system of the overhangs was developed and organized in a general scaffold (Fig. 1). Due to extensive character of the envisioned expression cassettes, each of the overhangs was assigned a letter, to enable unambiguous referencing to the respective position in the GGA. Moreover, the overhangs flanking individual genes (in position G1, G2 or G3; see Fig. 1) were designed to minimize the scar between a coding sequence and the regulatory elements. Thus, the overhang prior to the coding sequence was designed to maximally fit into the consensus sequence preceding significant fraction of ORFs in *Y. lipolytica* genome – CACA – or include the start codon (Gasmi *et al*., 2011).

The GG modular system was designed to include two flanking insertion sites, a selection marker and one, two or three TUs in each assembly. The number of TUs can be easily manipulated by amplification of ‘hybrid’ terminators, provided that the same sequence was used in the merged positions (T1, T2, T3; compare Fig. 1). These ‘hybrid’ terminators should be amplified with forward primer with ‘E’ overhang and reverse primer with ‘L’ overhang to render T1‐3 ‘hybrid’ terminator, skipping the second and third transcription units, or with ‘H’ overhang bearing forward primer and ‘L’ overhang bearing reverse primer, to gain T2‐3 ‘hybrid’ terminator, skipping the third transcription unit.

The destination vector backbones were equipped with a chromophore (RFP or amilCP) or fluorophore (amilGFP) gene flanked with BsaI sites with predesigned overhangs to define a colour‐based negative cloning selection marker. The BsaI recognition sites were positioned outwardly with respect to the backbone of the destination vector, so that after digestion with BsaI, both the reporter gene and the BsaI recognition sites were released, leaving the vector's backbone flanked with protruding 4 nt overhangs. A set of different antibiotic resistance genes was incorporated into the system of the destination vectors, to enable greater flexibility of experimental set‐up. In the proof‐of‐concept experiments (with three TUs bearing cassette; described below), we have used a pSB1K3‐RFP variant from the iGEM collection (http://parts.igem.org/Collections).

It is commonly accepted that only integrative expression cassettes constitute truly potent tools in *Y. lipolytica* genetic engineering, as the so far developed episomal plasmids tend to be unstable and low‐copy (Liu *et al*., 2014). This fact ruled the construction of both the destination plasmids as well as the scaffold of GGA, flanked with GGFs targeting integration of the expression cassette into the host genome (InsUP and InsDOWN). Thus, the destination vector's backbone containing solely bacterial elements can be discarded prior to transformation of *Y. lipolytica* cells. To enable the release of the expression cassette composed of transcription units, selection marker and targeting sequences, from the bacterial backbone, rare‐cutting restriction endonuclease recognition sites NotI or SfiI, depending on the targeted GGA structure, were added at the borders of the expression cassette in the InsUP and InsDOWN fragments (Fig. 1).

### Generation of GG library dedicated to *Y. lipolytica*

All the sequences to be used as building blocks of the envisioned GGA were extracted from *Y. lipolytica* W29 genome sequence or one of the previously constructed vectors from our own collection. All the sequences were analysed *in silico* to find internal BsaI site, to be eventually eliminated using assembly PCR technique (Sambrook and Russell, 2001). Subsequently, an extensive set of primers (referred to as GGP, for Golden Gate Primers) equipped with the predesigned system of the 4 nt overhangs and externally located BsaI recognition sites was designed (Supporting Information; Pauthenier and Faulon, 2014). The set of GGF presented in this work, the interchangeable parts for promoter, terminator, selection marker and integration site (Fig. 1B), constitutes the response to our current needs, but can be easily expanded on the other building blocks of interest by simply adding the required prefixes and suffixes within the primers at the stage of the element amplification. As presented in Fig. 1, the major focus of this study was to develop a system of easily tractable phenotype of positive transformants. Therefore, for the genes of interest we have chosen the complete pathway for carotenoid production, for convenient traceability of positive clones. With the same concept in mind, *LIP2* and *URA3* loci were selected as the targeting platforms, and thus, correct integration could be verified on tributyrin‐ or sucrose‐containing plate respectively. A *LIP2* deletion results in a reduced halo of triglyceride hydrolysis (Pignède *et al*., 2000), while replacement of *ura3‐320* allele bearing *S. cerevisiae SUC2* gene gives transformants unable to grow on sucrose (Nicaud *et al*., 1989; Barth and Gaillardin, 1996). Zeta sequences were chosen to reach random integrations in Po1d strain (JMY195) or at a zeta docking platform in Po1d derivative JMY1212, bearing a zeta sequence at the *leu2‐270* locus (Bordes *et al*., 2007).

All the amplicons bearing respective GGFs were cloned in the donor vectors (pCR Blunt II TOPO vectors; Thermo Fisher Scientific, Villebon sur Yvette, France), verified by BsaI digestions and sequencing and deposited in our collection. The structure of the targeted GGA cassette ruled the composition of the GG reaction mixture. The Golden Gate reaction conditions were designed based on the previously published protocols (Engler *et al*., 2008; Pauthenier *et al*., 2012; Agmon *et al*., 2015). The reaction mixture contained precalculated equimolar amount of each GGF and the destination vector (50 pmoles of ends), 2 μl of T4 DNA ligase buffer (NEB), 5 U of BsaI, 200 U of T4 and ddH2O up to 20 μl. The following thermal profile was applied: [37°C for 5 min, 16°C for 2 min] × 60, 55°C for 5 min, 80°C for 5 min, 15°C ∞. Subsequently, the reaction mixture was used for *E. coli* DH5α transformation (Sambrook and Russell, 2001). White colonies were screened for identification of complete GGA through plasmid isolation, restriction digestion and multiplex PCR. Complete GGA was subsequently linearized and used for transformation of either *Y. lipolytica* Po1d *ura‐ leu‐* (JMY195 *MatA leu2‐270 ura3‐302 xpr2‐322*) or Po1d *ura‐ leu+* bearing a zeta docking platform (JMY1212; *MatA ura3‐302 xpr2‐322, LEU2, zeta*; Bordes *et al*., 2007) via the lithium acetate transformation protocol (Chen *et al*., 1997). Derivative strain Po1d *leu+ ura+* (JMY2900; *MatA ura3‐302 xpr2‐322*) was used as prototroph control. Clones bearing complete GGA integrated with the host genome exhibited red–orange phenotype due to carotenoid production. All the GGF building blocks included in the GGA have been proven functional in previous experiments (see Table S1).

### Proof of concept based on carotenoid‐pathway‐containing Golden Gate Assembly

**Figure 2:**
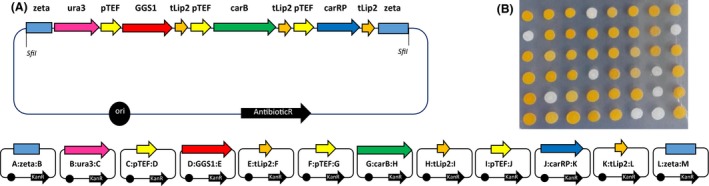
Golden Gate Assembly bearing ‘carotenoid synthesis’ pathway dedicated to integration with *Yarrowia lipolytica* genome. A. Scaffold of GGA comprising three transcription units and selection marker, flanked with integration targeting sequences, constructed on a destination vector backbone. Each gene was flanked with 396 nt of TEF promoter and 122 nt of Lip2 terminator sequences, both native to *Y. lipolytica*. *URA3* (1289 nt) gene was used as selection marker in this assembly. Random integrations in Po1d strain (JMY195) were driven through zeta sequences (305 nt and 395 nt for UP and DOWN respectively). Representation of donor vectors bearing respective GGFs is provided below. B. *Y. lipolytica* JMY195 transformants bearing ‘carotenoid synthesis’ GGA. Variability in colour development is discussed in the Results and Discussion section.

To validate robustness and efficiency of the here developed GG system, we have constructed a complex assembly structure comprising twelve GGFs and the destination vector, covering three TUs, accompanied by selection marker and integration targeting sequences (Fig. 2A).

The three genes within this assembly were native GGS1 (geranylgeranyl diphosphate synthase; YALI0D17050g) in G1 position and heterologous carB (phytoene dehydrogenase; AJ238028.1) and carRP (phytoene synthase; AJ250827.1) coding sequences from *Mucor circinelloides* in G2 and G3 positions respectively (sequences were kindly provided by DSM Company). Such a set of genes was previously proved to be efficiently expressed in *Y. lipolytica* cells, constructed using DNA assembler strategy (Gao *et al*., 2014) or classical cloning techniques (Matthaus *et al*., 2014). All the genes were assembled with corresponding regulatory elements, zeta insertion sites and *URA3* selection marker. The correct complete GGA was first preselected in *E. coli* clones through multiplex PCR and restriction digestion and then linearized and transformed into competent *Y. lipolytica* JMY195 cells. On average, more than 4 × 10^2^ colonies could be obtained from a single transformation reaction. About 10% of the transformants were white (exemplary results for one transformation run: 50/517, 91/1052, 41/396). The remaining clones exhibited different intensity of the orange colour development (Fig. 2B). The observed variability in the carotenoid‐producer phenotype could result from varying number of either complete or partial GGA copies integrated within the host genome as observed by Gao *et al*. (2014), although this would be unlikely with the amount of DNA we used (Bordes *et al*., 2007). More likely, the random distribution of the cassette within the genomic DNA due to the zeta targeting sequences, as investigated in Pignede *et al*. (2000), may be responsible for the colour variability. This hypothesis is supported by the results obtained when the same GGA was used to transform strain JMY1212, bearing a zeta docking platform at the leu2‐270 locus enabling homologous recombination. Although the number of white colonies after transformation of this strain was higher (33%; 33/98), the orange colonies did not present the colour variability that was found amongst JMY195‐based transformants. In a previous study with DNA assembler strategy, when the same carotenoid‐pathway genes were used, the efficiency of reaching desired phenotype was 20% (Gao *et al*., 2014). In the current study, the efficiency was largely improved, as 67% to 90% of the obtained *Y. lipolytica* clones exhibited desired red–orange phenotype, for JMY1212 and JMY195 strains respectively. Improved efficiency was accompanied by time‐ and workload savings, as well as versatility and standardization, being indigenous for modular DNA cloning techniques, altogether making this approach a method of choice for synthetic biology endeavours.

Here, we present a Golden Gate standard specifically designed to modify *Y. lipolytica* in a versatile manner. Robustness and reliability of the system were validated for thirteen‐element‐bearing GGA, with high efficiency of the desired phenotype recovery in the recombinants. This technology greatly enriches the molecular biology toolbox dedicated to this industrially relevant microorganism, permitting faster and accurate multiple target engineering, a limitless expansion of the building blocks library from endogenous or heterologous origins, or the use of interchangeable modules in combinatorial approaches followed by screening desired phenotypes.

## Authors' contribution

EC and RLA contributed equally to this work. EC, RLA, ML and TR contributed experimental data. CP participated in the selection of the 4 nt overhangs for the GGA scaffold and provided destination vectors. EC, RLA and JMN wrote the manuscript. The work was performed in the laboratory of TR and JMN.

## Conflict of interest

The authors do not have any conflict of interest.

## Supporting information


**Table S1.** A list of plasmids and oligonucleotides used in this study.
