# Supplementary material for: Golden Gate Assembly system dedicated to complex pathway manipulation in Yarrowia lipolytica
Source: Microb Biotechnol. 2017 Feb 19;10(2):450–5. doi: 10.1111/1751-7915.12605 (PMC5328822; doi:10.1111/1751-7915.12605)
Supplement: Supplementary file 1 — Table S1. A list of plasmids and oligonucleotides used in this study. [file MBT2-10-450-s001.docx]

| **PLASMIDS** | | | |
| --- | --- | --- | --- |
| ***Name*** | ***Resistance*** | ***Function (reporter)*** | ***Reference**** |
| pSB1K3-RFP | kanamycin | Destination vector (RFP – red chromophore) | (http://parts.igem.org/Collections). |
| pSB1C3-RFP | chloramphenicol |  |  |
| pSB1A3-RFP | ampicilin |  |  |
| pSB1K3-AmilGFP | kanamycin | Destination vector (AmylGFP – green fluorescence) |  |
| pSB1C3-AmilGFP | chloramphenicol |  |  |
| pSB1A3-AmilGFP | ampicilin |  |  |
| pSB1K3-AmilCP | kanamycin | Destiantion vector (AmylCP – blue) |  |
| pSB1C3-AmilCP | chloramphenicol |  |  |
| pSB1A3-AmilCP | ampicilin |  |  |
| pCR™-Blunt II-TOPO® | kanamycin | Donor vector | |
|  | | | |
| **OLIGONUCLEOTIDES** | | | |
| ***Name*** | ***Sequence*** | | ***Reference***** |
| **Amplification of GGFs** | | | |
| GGP_InsertUP_zeta_A_F_Not | GGTCTCTGCCTGCGGCCGCtgtcgggaaccgc | | (Bordes *et al.*, 2007) |
| GGP_InsertUP_zeta_A_F_SfiI | GGTCTCtGCCTggccacctaggcctgtcgggaaccgc | |  |
| GGP_InsertUP_zeta_B_R | GGTCTCTACCTtctagcaaagtgctttgtgc | |  |
| GGP_InsertUP_pMfe2_A_F_Not | GGTCTCtGCCTGCGGCCGCTTGTGTGTGTGTGTTGAATAAATAG | | (Dulermo and Nicaud, 2011) |
| GGP_InsertUP_ pMfe2_B_R | GGTCTCtACCTTCTGAACAATTGAGGACCC | |  |
| GGP_InsertUP_pLIP2_A_F_Not | GGTCTCtGCCTGCGGCCGCCGATGTCGATATCACTGACC | | (Pignède *et al.*, 2000) |
| GGP_InsertUP_pLIP2_B_R | GGTCTCtACCTAGTGAGGGGAGCTGG | |  |
| GGP_InsertUP_pURA3_A_F_Not | GGTCTCtGCCTGCGGCCGCGTCGACGAGTATCTGTCTG | | (Le Dall *et al.*, 1994) (Nicaud *et al.*, 2002) |
| GGP_InsertUP_pURA3_B_R | GGTCTCtACCTTTTGGTGGTGAAGAGGAG | |  |
| GGP_M_Ura3_B_F | GGTCTCTAGGTataacttcgtatagcatacattatacgaag | | (Le Dall *et al.*, 1994) (Nicaud *et al.*, 1989) |
| GGP_M_Ura3_C_R | GGTCTCTACGGtcgcttcggataactcc | |  |
| GGP_M_Lys5_B_F | GGTCTCtAGGTattaccctgttatccctacataacttcgtatagcat | | (Xuan *et al.*, 1990) |
| GGP_M_Lys5_C_R | GGTCTCTACGGtaggatatcccggatccgtgggaa | |  |
| GGP_P1_TEF_C_F | GGTCTCTACGGgacgggttggcgg | | (Müller *et al.*, 1998) (Ledesma-Amaro *et al.*, 2015) |
| GGP_P1_TEF_D_R | GGTCTCTCATTtgattcttatactcagaaggaaatgc | |  |
| GGP_P1_PGM_C_F | GGTCTCTACGGtaccaaccacagattacgac | | (Picataggio and Zhu, 2008) |
| GGP_P1_PGM_D_R | GGTCTCTCATTttttgtatgtgttttggtgatgtc | |  |
| GGP_P1_GAPdh_C_F | GGTCTCTACGGcggtagtcggaaagagc | | (Picataggio and Zhu, 2008) |
| GGP_P1_GAPdh_D_R | GGTCTCTCATTtgttgatgtgtgtttaattcaagaatg | |  |
| GGP_P1_2UAS_TEF_C_F | GGTCTCtACGGCGATACGCGTAtcgatacgc | | (Nicaud *et al.*, 2002) (Blazeck *et al.*, 2011) |
| GGP_P1_2UAS_TEF_D_R | GGTCTCtCATTggatccttcg | |  |
| GGP_P1_4UAS_TEF_C_F | GGTCTCtACGGCGATACGCGTatcgatacgc | |  |
| GGP_P1_4UAS_TEF_D_R | GGTCTCtCATTggatccttcg | |  |
| GGP_P1_8UAS_TEF_C_F | GGTCTCtACGGCGATACGCGTatcgatgatac | |  |
| GGP_P1_8UAS_TEF_D_R | GGTCTCtCATTggatccttc | |  |
| GGP_P1_4UAS_TEF_Leu2_C_F | GGTCTCtACGGCGATACGCGT | |  |
| GGP_P1_4UASpLeu2_D_R | GGTCTCtCATTCTTAGTTTCGGGTTCCCAC | |  |
| GGP_P1_4UASpTEF_D_R | GGTCTCtCATTCTTCGGGTGTGAGTTGAC | |  |
| GGP_G1_GGS1_D_F | GGTCTCTAatggattataacagcgcgg | | (Gao *et al.*, 2014) |
| GGP_G1_GGS1_E_R | GGTCTCTTAGAtcactgcgcatcctc | |  |
| GGP_T1_LIP2_E_F | GGTCTCTTCTAgtgtctgtggtatctaagctatttatc | | (Nicaud *et al.*, 2002) (Ledesma-Amaro *et al.*, 2015) |
| GGP_T1_LIP2_F_R | GGTCTCTAAGCcgatttgtcttagaggaacgc | |  |
| GGP_T1_TEF_E_F | GGTCTCTTCTAgctgcttgtacctagtgc | | (Celińska *et al.*, 2016) |
| GGP_T1_TEF_F_R | GGTCTCTAAGCttttttttttttttttaactcagaagtttttgac | |  |
| GGP_T1_XPR_E_F | GGTCTCtTCTAatcggcccgggc | | (Nicaud *et al.*, 2002) |
| GGP_T1_XPR_F_R | GGTCTCtAAGCaagttgctgcttgatggg | |  |
| GGP_T1_TGuo1_E_F | GGTCTCTTCTATATATAACTGTCTAGAAATAAAGAGTATCATCTTTCAAAGCTTAGAGACC | | (Curran *et al.*, 2015) |
| GGP_T1_ TGuo1_F_R | GGTCTCTAAGCTTTGAAAGATGATACTCTTTATTTCTAGACAGTTATATATAGAAGAGACC | |  |
| GGP_T1_Tsynth27_E_F | GGTCTCTTCTATGGGTGGTATATATATATATATATATATATATAACTGTCTAGAAATAAAGAGTATCATCTTTCAAAGCTTAGAGACC | |  |
| GGP_T1_ Tsynth27_F_R | GGTCTCTAAGCTTTGAAAGATGATACTCTTTATTTCTAGACAGTTATATATATATATATATATATATATACCACCCATAGAAGAGACC | |  |
| GGP_T1_Tsynth8_E_F | GGTCTCTTCTATATATAAACTCATTTACTTATGTAGGAATAAAGAGTATCATCTTTCAAAGCTTAGAGACC | |  |
| GGP_T1_ Tsynth8_F_R | GGTCTCTAAGCTTTGAAAGATGATACTCTTTATTCCTACATAAGTAAATGAGTTTATATATAGAAGAGACC | |  |
| GGP_P2_TEF_F_F | GGTCTCTGCTTgacgggttggcgg | | (Müller *et al.*, 1998) (Ledesma-Amaro *et al.*, 2015) |
| GGP_P2_TEF_G_R | GGTCTCTTTGTtgattcttatactcagaaggaaatgc | |  |
| GGP_P2_PGM_F_F | GGTCTCTGCTTtaccaaccacagattacgac | | (Picataggio and Zhu, 2008) |
| GGP_P2_PGM_G_R | GGTCTCTTTGTttttgtatgtgttttggtgatgtc | |  |
| GGP_P2_GAPdh_F_F | GGTCTCTGCTTcggtagtcggaaagagc | | (Picataggio and Zhu, 2008) |
| GGP_P2_GAPdh_G_R | GGTCTCTTTGTtgttgatgtgtgtttaattcaagaatg | |  |
| GGP_P2_2UAS_TEF_F_F | CCCGGTCTCtGCTTCGATACGCGTATCGATACGCG | | (Nicaud *et al.*, 2002) (Blazeck *et al.*, 2011) |
| GGP_ P2_2UAS_TEF_G_R | CCCGGTCTCtTTGTggatccttcgggtgtgagttg | |  |
| GGP_ P2_4UAS_TEF_F_F | CCCGGTCTCtGCTTCGATACGCGTATCGATACGCG | |  |
| GGP_ P2_4UAS_TEF_G_R | CCCGGTCTCtTTGTggatccttcgggtgtgagttg | |  |
| GGP_ P2_8UAS_TEF_F_F | CCCGGTCTCtGCTTCGATACGCGTatcgatgatacgc | |  |
| GGP_ P2_8UAS_TEF_G_R | CCCGGTCTCtTTGTggatccttcgggtgtgagttg | |  |
| GGP_P2_4UAS_pTEF_pLEU2_F_F | GGTCTCTGCTT CGATACGCGT | |  |
| GGP_P2_4UASpLEU2_G_R | GGTCTCTTTGTCTTAGTTTCGGGTTCCCAC | |  |
| GGP_P2_4UASpTEF_G_R | GGTCTCTTTGTCTTCGGGTGTGAGTTGAC | |  |
| GGP_G2_carB_G_F | GGTCTCTACAAtgtccaagaaacacattgtcattatc | | (Gao *et al.*, 2014) |
| GGP_G2_carB_H_R | GGTCTCTATCCttaaatgacattagagttatgaacgc | |  |
| GGP_T2_LIP2_H_F | GGTCTCTGGATgtgtctgtggtatctaagctatttatc | | (Nicaud *et al.*, 2002) (Ledesma-Amaro *et al.*, 2015) |
| GGP_T2_LIP2_I_R | GGTCTCTTGACcgatttgtcttagaggaacgc | |  |
| GGP_T2_TEF_H_F | GGTCTCTGGATgctgcttgtacctagtgc | | (Celińska *et al.*, 2016) |
| GGP_T2_TEF_I_R | GGTCTCTTGACttttttttttttttttaactcagaagtttttgac | |  |
| GGP_T2_XPR_H_F | GGTCTCTGGATATCGGCCCGGGC | | (Nicaud *et al.*, 2002) |
| GGP_T2_XPR_I_R | GGTCTCTTGACAAGTTGCTGCTTGATGGG | |  |
| GGP_T2_TGuo1_H_F | GGTCTCtGGATtatataactgtctagaaataaagagtatcatctttcaaaGTCAaGAGACC | | (Curran *et al.*, 2015) |
| GGP_T2_ TGuo1_I_R | GGTCTCtTGACtttgaaagatgatactctttatttctagacagttatataATCCaGAGACC | |  |
| GGP_T2_Tsynth27_H_F | GGTCTCtGGATtgggtggtatatatatatatatatatatatataactgtctagaaataaagagtatcatctttcaaaGTCAaGAGACC | |  |
| GGP_T2_ Tsynth27_I_R | GGTCTCtTGACtttgaaagatgatactctttatttctagacagttatatatatatatatatatatatataccacccaATCCaGAGACC | |  |
| GGP_T2_Tsynth8_H_F | GGTCTCtGGATtatataaactcatttacttatgtaggaataaagagtatcatctttcaaaGTCAaGAGACC | |  |
| GGP_T2_ Tsynth8_I_R | GGTCTCtTGACtttgaaagatgatactctttattcctacataagtaaatgagtttatataATCCaGAGACC | |  |
| GGP_P3_TEF_I_F | GGTCTCTGTCAgacgggttggcgg | | (Müller *et al.*, 1998) (Ledesma-Amaro *et al.*, 2015) |
| GGP_P3_TEF_J_R | GGTCTCTGTGGtgattcttatactcagaaggaaatgc | |  |
| GGP_P3_PGM_I_F | GGTCTCTGTCAtaccaaccacagattacgac | | (Picataggio and Zhu, 2008) |
| GGP_P3_PGM_J_R | GGTCTCTGTGGttttgtatgtgttttggtgatgtc | |  |
| GGP_P3_GAPdh_I_F | GGTCTCTGTCAcggtagtcggaaagagc | | (Picataggio and Zhu, 2008) |
| GGP_P3_GAPdh_J_R | GGTCTCTGTGGtgttgatgtgtgtttaattcaagaatg | |  |
| GGP_P3_2UAS_TEF_I_F | CCCGGTCTCtGTCACGATACGCGTATCGATACGCG | | (Nicaud *et al.*, 2002) (Blazeck *et al.*, 2011) |
| GGP_ P3_2UAS_TEF_J_R | CCCGGTCTCtGTGGggatccttcgggtgtgagttg | |  |
| GGP_ P3_4UAS_TEF_I_F | CCCGGTCTCTGTCACGATACGCGTATCGATACGCG | |  |
| GGP_ P3_4UAS_TEF_J_R | CCCGGTCTCtGTGGggatccttcgggtgtgagttg | |  |
| GGP_ P3_8UAS_TEF_I_F | CCCGGTCTCtGTCACGATACGCGTatcgatgatacg | |  |
| GGP_ P3_8UAS_TEF_J_R | CCCGGTCTCtGTGGggatccttcgggtgtgagttg | |  |
| GGP_P3_4UAS_pTEF_hp4d_I_F | GGTCTCTGTCA CGATACGCGT | |  |
| GGP_P3_4UAShp4d_J_R | GGTCTCTGTGGCTTAGTTTCGGGTTCCCAC | |  |
| GGP_P3_4UASpTEF_J_R | GGTCTCTGTGGCTTCGGGTGTGAGTTGAC | |  |
| GGP_G3_carRP_J_F | GGTCTCTCCACAatgctgctcacctacatg | | (Gao *et al.*, 2014) |
| GGP_G3_carRP_K_R | GGTCTCTGTATttaaatggtatttagatttctcatttttccc | |  |
| GGP_T3_LIP2_K_F | GGTCTCTGTATgtgtctgtggtatctaagctatttatc | | (Nicaud *et al.*, 2002) (Ledesma-Amaro *et al.*, 2015) |
| GGP_T3_LIP2_L_R | GGTCTCTACTCcgatttgtcttagaggaacgc | |  |
| GGP_T3_TEF_K_F | GGTCTCTGTATgctgcttgtacctagtgc | | (Celińska *et al.*, 2016) |
| GGP_T3_TEF_L_R | GGTCTCTACTCttttttttttttttttaactcagaagtttttgac | |  |
| GGP_T3_XPR_K_F | GGTCTCtGTATatcggcccgggc | | (Nicaud *et al.*, 2002) |
| GGP_T3_XPR_L_R | GGTCTCtACTCaagttgctgcttgatggg | |  |
| GGP_T3_TGuo1_K_F | GGTCTCtGTATtatataactgtctagaaataaagagtatcatctttcaaaGAGTaGAGACC | | (Curran *et al.*, 2015) |
| GGP_T3_ TGuo1_L_R | GGTCTCtACTCtttgaaagatgatactctttatttctagacagttatataGTACaGAGACC | |  |
| GGP_T3_Tsynth27_K_F | GGTCTCtGTATtgggtggtatatatatatatatatatatatataactgtctagaaataaagagtatcatctttcaaaGAGTaGAGACC | |  |
| GGP_T3_ Tsynth27_L_R | GGTCTCtACTCtttgaaagatgatactctttatttctagacagttatatatatatatatatatatatataccacccaGTACaGAGACC | |  |
| GGP_T3_Tsynth8_K_F | GGTCTCtGTATtatataaactcatttacttatgtaggaataaagagtatcatctttcaaaGAGTaGAGACC | |  |
| GGP_T3_ Tsynth8_L_R | GGTCTCtACTCtttgaaagatgatactctttattcctacataagtaaatgagtttatataGTACaGAGACC | |  |
| GGP_InsertDOWN_zeta_L_F | GGTCTCTGAGTcatgtgtaacactcgctctg | | (Bordes *et al.*, 2007) |
| GGP_InsertDOWN_zeta_M_R_Not | GGTCTCTCGCAGCGGCCGCactgaagggctttgtgag | |  |
| GGP_InsertDOWN_zeta_M_R_SfiI | GGTCTCTCGCAGGCCTCCTTGGCCACTGAAGGGCTTTGTGAG | |  |
| GGP_InsertDown_pMfe2_L_F | GGTCTCtGAGTAACATTATATTGGGGGAGGG | | (Dulermo and Nicaud, 2011) |
| GGP_InsertDOWN_ pMfe2_M_R_Not | GGTCTCtCGCAAGCTATTATCTGACCAAGTGATAC | |  |
| GGP_InsertDOWN_pLIP2_L_F | GGTCTCtGAGTGCTATTTATCACTCTTTACAACTTCTAC | | (Pignède *et al.*, 2000) |
| GGP_InsertDOWN_pLIP2_M_R_Not | GGTCTCtCGCAGCGGCCGCACATCAGTATCCAAGGCAC | |  |
| GGP_InsertDOWN_pURA3_L_F | GGTCTCtGAGTAGGTTAGACTATGGATATGTCATTTAAC | | (Le Dall *et al.*, 1994) (Nicaud *et al.*, 2002) |
| GGP_InsertDOWN_pURA3_M_R_Not | GGTCTCtCGCAGCGGCCGCATAAGTATAAGTAGTTCAATCGTATTAGCTAC | |  |
|  | | | |
| **Elimination of internal BsaI site through assembly PCR** | | | |
| *GGS1_Bsa_del_F* | *GAAATAgtctcgagatcgctcttg* | | |
| *GGS1_Bsa_del_R* | *GAGACTatttcgcacatcaccaag* | | |
|  | | | |
| **Larger synthetic elements** | | | |
| Synthetic M (marker element) Leu2 | | | (Madzak *et al.*, 2000) |
| GGTCTCTAGGTATTACCCTGTTATCCCTACATAACTTCGT ATAGCATACATTATACGAAGTTATTCTGAATTCCGCCTGAGTCAT CATTTATTTACCAGTTGGCCACAAACCCTTGACGATCTCGTATGT CCCCTCCGACATACTCCCGGCCGGCTGGGTACGTTCGATAGCGCT ATCGGCATCGACAAGGTTTGGGTCCCTAGCCGATACCGCACTACC TGAGTCACAATCTTCGGAGGTTTAGTCTTCCACATAGCACGGGCA AAAGTGCGTATATATACAAGAGCGTTTGCCAGCCACAGATTTTCA CTCCACACACCACATCACACATACAACCACACACATCCACAATGG AACCCGAAACTAAGAAGACCAAGACTGACTCCAAGAAGATTGTTC TTCTCGGCGGCGACTTCTGTGGCCCCGAGGTGATTGCCGAGGCCG TCAAGGTGCTCAAGTCTGTTGCTGAGGCCTCCGGCACCGAGTTTG TGTTCGAGGACCGACTCATTGGAGGAGCTGCCATTGAGAAGGAGG GCGAGCCCATCACCGACGCTACTCTCGACATCTGCCGAAAGGCTG ACTCTATTATGCTCGGTGCTGTCGGAGGCGCTGCCAACACCGTAT GGACCACTCCCGACGGACGAACCGACGTGCGACCCGAGCAGGGCC TCCTCAAGCTGCGAAAGGACCTGAACCTGTACGCCAACCTGCGAC CCTGCCAGCTGCTGTCGCCCAAGCTCGCCGATCTCTCCCCCATCC GAAACGTTGAGGGCACCGACTTCATCATTGTCCGAGAGCTCGTCG GAGGTATCTACTTTGGAGAGCGAAAGGAGGATGACGGATCTGGCG TCGCTTCCGACACCGAGACTTACTCCGTTCCTGAGGTTGAGCGAA TTGCCCGAATGGCCGCCTTCCTGGCCCTTCAGCACAACCCCCCTC TTCCCGTGTGGTCCCTTGACAAGGCCAACGTGCTGGCCTCCTCTC GACTTTGGCGAAAGACTGTCACCCGAGTCCTCAAGGACGAGTTCC CCCAGCTGGAGCTCAACCACCAGCTGATCGACTCGGCCGCCATGA TCCTCATCAAGCAGCCCTCCAAGATGAATGGTATCATCATCACCA CCAACATGTTTGGCGATATCATCTCCGACGAGGCCTCCGTCATCC CCGGTTCTCTGGGTCTGCTGCCCTCCGCCTCTCTGGCTTCTCTGC CCGACACCAACGAGGCGTTCGGTCTGTACGAGCCCTGTCACGGAT CTGCCCCCGATCTCGGCAAGCAGAAGGTCAACCCCATTGCCACCA TTCTGTCTGCCGCCATGATGCTCAAGTTCTCTCTTAACATGAAGC CCGCCGGTGACGCTGTTGAGGCTGCCGTCAAGGAGTCCGTCGAGG CTGGTATCACTACCGCCGATATCGGAGGCTCTTCCTCCACCTCCG AGGTCGGAGACTTGTTGCCAACAAGGTCAAGGAGCTGCTCAAGAA GGAGTAAGTCGTTTCTACGACGCATTGATGGAAGGAGCAAACTGA CGCGCCTGCGGGTTGGTCTACCGGCAGGATCTGCTAGTGTATAAG ACTCTATAAAAAGGGCCCTGCCCTGCTAATGAAATGATGATTTAT AATTTACCGGTGTAGCAACCTTGACTAGAAGAAGCAGATTGGGTG TGTTTGTAGTGGAGGACAGTGGTACGTTTTGGAAACAGTCTTCTT GAAAGTGTCTTGTCTACAGTATATTCACTCATAACCTCAATAGCC AAGGGTGTAGTCGGTTTATTAAAGGAAGGGAGTTGTGGCTGATGT GGATAGATATCTTTAAGCTGGCGACTGCACCCAACGAGTGTGGTG GTAGCTTGTTACTGTATATTCGAATTCGTATAACTTCGTATAGCA GGAGTTATCCGAAGCGATAATTACCCTGTTATCCCTAGAGCTAGA GACC | | | |
| Synthetic M (marker element) Hyg | | | (Fickers *et al.*, 2003) |
| GGTCTCTAGGTATTACCCTGTTATCCCTACATAACTTCGT  ATAGCATACATTATACGAAGTTATTCTGAATTCGAGCACCGCCGC  CGCAAGGAATGGTGCATGCTGAGGTGTCTCACAAGTGCCGTGCAG  TCCCGCCCCCACTTGCTTCTCTTTGTGTGTAGTGTACGTACATTA  TCGTGACGGTTGTTCCCGCCCACCTCGATCCGGCATGCTGAGGTG  TCTCACAAGTGCCGTGCAGTCCCGCCCCCACTTGCTTCTCTTTGT  GTGTAGTGTACGTACATTATCGTGACGGTTGTTCCCGCCCACCTC  GATCCGGCATGCTGAGGTGTCTCACAAGTGCCGTGCAGTCCCGCC  CCCACTTGCTTCTCTTTGTGTGTAGTGTACGTACATTATCGTGAC  GGTTGTTCCCGCCCACCTCGATCCGGCATGCTGAGGTGTCTCACA  AGTGCCGTGCAGTCCCGCCCCCACTTGCTTCTCTTTGTGTGTAGT  GTACGTACATTATCGCGCCCGTTGTTCCCGCCCACCTCGATCCGG  CATGCACTGATCACGGGCAAAAGTGCGTATATATACAAGAGCGTT  TGCCAGCCACAGATTTTCACTCCACACACCACATCACACATACAA  CCACACACATCCACAATGAAAAAGCCTGAACTCACCGCCACGTCT  GTCGAGAAGTTTCTGATCGAAAAGTTCGACAGCGTCTCCGACCTG  ATGCAGCTCTCGGAGGGCGAAGAATCTCGTGCTTTCAGCTTCGAT  GTAGGAGGGCGTGGATATGTCCTGCGGGTAAATAGCTGCGCCGAT  GGTTTCTACAAAGATCGTTATGTTTATCGGCACTTTGCATCGGCC  GCGCTCCCGATTCCGGAAGTGCTTGACATTGGGGAATTTAGCGAG  AGCCTGACCTATTGCATCTCCCGCCGTGCACAGGGTGTCACGTTG  CAAGACCTGCCTGAAACCGAACTGCCCGCTGTTCTGCAGCCGGTC  GCGGAGGCCATGGATGCGATCGCTGCGGCCGATCTTAGCCAGACG  AGCGGGTTCGGCCCATTCGGACCGCAAGGAATCGGTCAATACACT  ACATGGCGTGATTTCATATGCGCGATTGCTGATCCCCATGTGTAT  CACTGGCAAACTGTGATGGACGACACCGTCAGTGCGTCCGTCGCG  CAGGCTCTCGATGAGCTGATGCTTTGGGCCGAGGACTGCCCCGAA  GTCCGGCACCTCGTGCACGCGGATTTCGGCTCCAACAATGTCCTG  ACGGACAATGGCCGCATAACAGCGGTCATTGACTGGAGCGAGGCG  ATGTTCGGGGATTCCCAATACGAGGTCGCCAACATCTTCTTCTGG  AGGCCGTGGTTGGCTTGTATGGAGCAGCAGACGCGCTACTTCGAG  CGGAGGCATCCGGAGCTTGCAGGATCGCCGCGGCTCCGGGCGTAT  ATGCTCCGCATTGGTCTTGACCAACTCTATCAGAGCTTGGTTGAC  GGCAATTTCGATGATGCAGCTTGGGCGCAGGGTCGATGCGACGCA  ATCGTCCGATCCGGAGCCGGGACTGTCGGGCGTACACAAATCGCC  CGCAGAAGCGCGGCCGTCTGGACCGATGGCTGTGTAGAAGTACTC  GCCGATAGTGGAAACCGACGCCCCAGCACTCGTCCGAGGGCAAAG  GAATAGTCGAGAATTCGTATAACTTCGTATAGCAGGAGTTATCCG  AAGCGATAATTACCCTGTTATCCCTAGAGCTAGAGACC | | | |

* Reference to Registry of Standard Biological Parts resource

** Study where operability of the sequence was validated in *Y. lipolytica*

**References**

Blazeck, J., Liu, L., Redden, H., and Alper, H. (2011) Tuning gene expression in yarrowia lipolytica by a hybrid promoter approach. *Appl. Environ. Microbiol.* **77**: 7905–7914.

Bordes, F., Fudalej, F., Dossat, V., Nicaud, J.-M., and Marty, A. (2007) A new recombinant protein expression system for high-throughput screening in the yeast Yarrowia lipolytica. *J. Microbiol. Methods* **70**: 493–502.

Celińska, E., Borkowska, M., and Białas, W. (2016) Evaluation of heterologous α-amylase production in two expression platforms dedicated for Yarrowia lipolytica: commercial Po1g-pYLSC (php4d) and custom-made A18-pYLTEF (pTEF). *Yeast* **33**: 165–181.

Curran, K.A., Morse, N.J., Markham, K.A., Wagman, A.M., Gupta, A., and Alper, H.S. (2015) Short Synthetic Terminators for Improved Heterologous Gene Expression in Yeast. *ACS Synth. Biol.* **4**: 824–832.

Le Dall, M.T., Nicaud, J.M., and Gaillardin, C. (1994) Multiple-copy integration in the yeast Yarrowia lipolytica. *Curr. Genet.* **26**: 38–44.

Dulermo, T. and Nicaud, J.M. (2011) Involvement of the G3P shuttle and Β-oxidation pathway in the control of TAG synthesis and lipid accumulation in Yarrowia lipolytica. *Metab. Eng.* **13**: 482–491.

Fickers, P., Le Dall, M.T., Gaillardin, C., Thonart, P., and Nicaud, J.M. (2003) New disruption cassettes for rapid gene disruption and marker rescue in the yeast Yarrowia lipolytica. *J. Microbiol. Methods* **55**: 727–737.

Gao, S., Han, L., Zhu, L., Ge, M., Yang, S., Jiang, Y., and Chen, D. (2014) One-step integration of multiple genes into the oleaginous yeast Yarrowia lipolytica. *Biotechnol. Lett.* **36**: 2523–2528.

Ledesma-Amaro, R., Dulermo, T., and Nicaud, J.M. (2015) Engineering Yarrowia lipolytica to produce biodiesel from raw starch. *Biotechnol. Biofuels* **8**: 148.

Madzak, C., Tréton, B., and Blanchin-Roland, S. (2000) Strong hybrid promoters and integrative expression/secretion vectors for quasi-constitutive expression of heterologous proteins in the yeast Yarrowia lipolytica. *J. Mol. Microbiol. Biotechnol.* **2**: 207–216.

Müller, S., Sandal, T., Kamp-Hansen, P., and Dalbøge, H. (1998) Comparison of expression systems in the yeasts Saccharomyces cerevisiae, Hansenula polymorpha, Klyveromyces lactis, Schizosaccharomyces pombe and Yarrowia lipolytica. Cloning of two novel promoters from Yarrowia lipolytica. *Yeast* **14**: 1267–1283.

Nicaud, J., Fabre, E., and Gaillardin, C. (1989) Expression of invertase activity in Yarrowia lipolytica and its use as a selective marker. *Curr. Genet.* **16**: 253–260.

Nicaud, J.M., Madzak, C., van den Broek, P., Gysler, C., Duboc, P., Niederberger, P., and Gaillardin, C. (2002) Protein expression and secretion in the yeast Yarrowia lipolytica. *FEMS Yeast Res.* **2**: 371–379.

Picataggio, S.K. and Zhu, Q.Q. (2008) Glyceraldehyde-3-phosphate dehydrogenase and phosphoglycerate mutase regulatory sequences for gene expression in oleaginous yeast.

Pignède, G., Wang, H., Fudalej, F., Gaillardin, C., Seman, M., and Nicaud, J.M. (2000) Characterization of all extracellular lipase encoded by LIP2 in Yarrowia lipolytica. *J. Bacteriol.* **182**: 2802–2810.

Xuan, J.W., Fournier, P., Declerck, N., Chasles, M., and Gaillardin, C. (1990) Overlapping Reading Frames at the LYS5 Locus in the Yeast Yarrowia lipolytica. *Mol. Cell. Biol.* **10**: 4795.
